# A machine learning classifier approach for identifying the determinants of under-five child undernutrition in Ethiopian administrative zones

**DOI:** 10.1186/s12911-021-01652-1

**Published:** 2021-10-24

**Authors:** Haile Mekonnen Fenta, Temesgen Zewotir, Essey Kebede Muluneh

**Affiliations:** 1 Department of Statistics, College of Science, Bahir Dar University, Bahir Dar, Ethiopia; 2 School of Mathematics, Statistics and Computer Science, College of Agriculture Engineering and Science, University of KwaZulu-Natal, Durban, South Africa; 3 School of Public Health, College of Medicine and Health Sciences, Bahir Dar University, Bahir Dar, Ethiopia

**Keywords:** Composite index for anthropometric failure (CIAF), Confusion matrix, Covariate selection and ranking, Multicollinearity, Receiver operating characteristics (ROC)

## Abstract

**Background:**

Undernutrition is the main cause of child death in developing countries. This paper aimed to explore the efficacy of machine learning (ML) approaches in predicting under-five undernutrition in Ethiopian administrative zones and to identify the most important predictors.

**Method:**

The study employed ML techniques using retrospective cross-sectional survey data from Ethiopia, a national-representative data collected in the year (2000, 2005, 2011, and 2016). We explored six commonly used ML algorithms; Logistic regression, Least Absolute Shrinkage and Selection Operator (L-1 regularization logistic regression), L-2 regularization (Ridge), Elastic net, neural network, and random forest (RF). Sensitivity, specificity, accuracy, and area under the curve were used to evaluate the performance of those models.

**Results:**

Based on different performance evaluations, the RF algorithm was selected as the best ML model. In the order of importance; urban–rural settlement, literacy rate of parents, and place of residence were the major determinants of disparities of nutritional status for under-five children among Ethiopian administrative zones.

**Conclusion:**

Our results showed that the considered machine learning classification algorithms can effectively predict the under-five undernutrition status in Ethiopian administrative zones. Persistent under-five undernutrition status was found in the northern part of Ethiopia. The identification of such high-risk zones could provide useful information to decision-makers trying to reduce child undernutrition.

**Supplementary Information:**

The online version contains supplementary material available at 10.1186/s12911-021-01652-1.

## Background

Proper nutrition is so crucial to lead a healthy lifestyle. Malnutrition, particularly undernutrition, is a global concern for the health condition and survival of children [1–5]. Almost half of the deaths of children in developing countries were directly or indirectly linked to malnutrition [3, 6]. Malnourished children are more vulnerable to different illnesses compared to their counterparts [1–6]. A considerable number of studies investigating the issue targeting under-five children malnutrition and the risk factors associated with this age group. These studies employed classical models such as generalized linear (mixed) models [4, 5, 7–10]. The finding from the investigations, among others, showed that the nutritional status of children of this age group has gradually improved over the last 2 decades in Ethiopia. Particularly, it has been found that the prevalence of under-five children underweight in Ethiopia was 47.1% in 2000, 38.5% in 2005, 28.8% in 2011, 23.3 in 2016, and 20.56% in 2019, while the prevalence of stunting was 51.22% in 2000, 46.5% in 2005, 44.3% in 2011, 38.3% in 2016, and 36.9% in 2019. Similarly, 10.7% of under-five children were wasted in 2000, 10.5% in 2005, 9.9% in 2011, 10.1% in 2016, and 7% in 2019. The prevalence of having at least one of the undernutrition indicators measured in terms of the composite index for anthropometric failure (CIAF) was 61.38% in 2000, 56.58% in 2005, 51.58% in 2011, 46.49% in 2016, and 42.4 in 2019. Moreover, the CIAF is computed by grouping different forms of anthropometric failure as such: B-wasting only, C-wasting and underweight, D-wasting, stunting and underweight, E-stunting and underweight, F-stunting only, and Y-underweight only. The CIAF, calculated by aggregating these six (B–Y) categories [11–15]. Most of such studies conducted in this country depicted the effects of socio-economic and demographic covariates that were associated with under-five children undernutrition status using the classical regression models [4, 5, 7, 8]. Those traditional models are widely used for causal inferences and with the selection of built-in features, with a relatively small number of covariates [16, 17]. Correlations between covariates (multicollinearity) and a large number of factors are the common analytical challenges in traditional modeling [18–21]. Moreover, as compared to those classical models, the machine learning (ML) methods have the qualities of using a larger number of predictors, requiring fewer assumptions, incorporating “multi-dimensional correlations”, and producing a more flexible relationship among the predictor variables and the outcome variables [16–18, 20–22]. In addition, the ML models can create models for prediction purposes that show superiority in taking care of classification problems when compared with the classical approaches [16–18, 21, 23]. In the present paper, we focused to predict CIAF in Ethiopia using this tool drawing on the nationally representative data. Machine learning employs methods developed within the disciplines of statistics, computer sciences, mathematics, and artificial intelligence which allow the formation of algorithms that can learn from and make predictions using data [24–29]. As such, it is applicable in different disciplines, such as in medical sciences; for diagnosis and outcome prediction [23, 30–44], disease modeling [33], disease prediction [34–37], child mortality [23, 38], and it is also used in industrial applications [39–41]. Just only a few studies had investigated the role of this tool to create prediction models of childhood for malnutrition [42–44]. Moreover, the study is conducted at the administrative zones in Ethiopia. This is because, in the country, the zonal health departments have the mandate to plan, follow up, monitor, and evaluate health activities of Woreda health offices and the different Woredas in the same Zone are relatively similar in many respects. Moreover, the administrative Zones are mainly ethnic-based, and the assessment of the Zones provides cultural practices regarding staple food and the geographic environment of the community in the Zones [45–48]. Hence detecting the problems of undernutrition and its variations among administrative Zones provides deeper insight into the health priorities which helps policymakers to design focused intervention strategies. The main objective of this study was, therefore, to identify ML algorithms in predicting and identifying the important covariates that underline the spatial variations in childhood CIAF among 72 Ethiopian administrative zones.

## Materials and methods

**Fig. 1:**
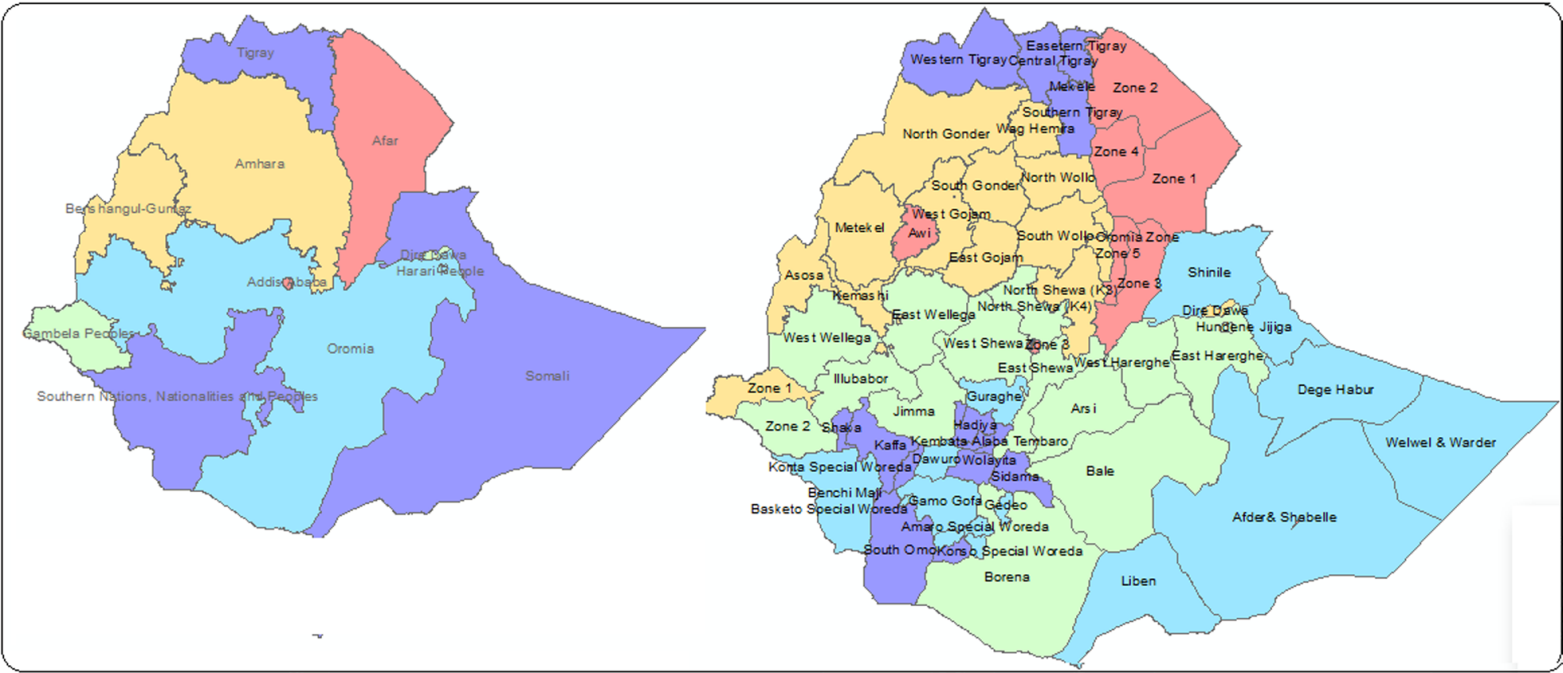
Map of Ethiopia with regions and zones: **A** the 11 regions of the country and **B** the 72 administrative zones

This study was carried out on the disparities of malnutrition in Ethiopia, with a surface area of 1.1 million km^2^, the country shares borders with Eritrea in the north, Djibouti and Somali in the east, Sudan and South Sudan in the west, and Kenya in the south. It is divided into 11 administrative units (regions) including Addis Ababa, the capital city of the country. The regions were further divided into 72 second-level administrative boundaries called zones [49] (Fig. 1).

### Data sources and analysis tools

We conducted the analysis based on the four EDHS datasets (2000, 2005, 2011, and 2016), a nationally representative household survey developed by the United States Agency for International Development (USAID) in the 1980s [50]. The outcome variable that we aimed to predict is the undernutrition status of under-five children measured in terms of the composite index for anthropometric failure (CIAF). CIAF is measured as a binary response as being nourished (coded as 0) and undernourished (coded as 1). The covariates (features) were collected from different pieces of literature [4, 5, 7–10]. All the categorical features are converted to numerical dummy variables, by mapping each unique value to a number [4, 5, 7–10]. The boundaries (shapes) were used to define the second-level administrative zones and merged with the real dataset for analysis [51].

### Methodology

*Model building* The ML models have shown superiority in taking care of classification problems when compared with the traditional models (like generalized linear mixed models). The raw data are usually not found in the form and shape that is required for optimal performance of the machine learning algorithms. The algorithms that would be implemented in ML are only numerical values and therefore it is important to transform the categorical variables into numerical values. Hence, the preprocessing step is the most important aspect in the ML model applications [21, 23, 52–54]. The categorical features of the dataset are encoded to transform these features into numerical values and the continuous data in this study were normalized. For ML approaches, the dataset is randomly split into two: a training dataset which trains the model, and a test dataset where we predict the response variable and check whether the predicted outcome is similar to the actual outcomes, and the validation dataset is considered for the parameter estimates to be incorporated in the training models [24–29]. Influence of different training and testing ratios on the performance of the given ML models were checked. This study (train/test: 80/20, and 70/30) was implemented to divide the datasets into the training and testing datasets for performance assessment of models. Popular statistical indicators have been employed to evaluate the predictive capability of the models under different training and testing ratios. The results revealed that the train-test 70–30% split were more advantageous to undernutrition classification than their counterparts (80/20). A variety of supervised ML algorithms including Logistic Regression (LR) [55], Ridge regression [56], Least Absolute Shrinkage and Selection Operator (LASSO) regression [57], Elastic Net [27, 58], Artificial Neural Network (ANN) [59, 60] and Random Forest (RF) [27, 61] were included in the analysis.

The Ridge, Lasso, and Elastic Net are very similar to LR, except that we have an additional penalty term called regularization to estimate the regression coefficients [26, 27] to reduce the over-fitting and the adverse effects of multicollinearity [26–28, 62]. The advantage of ridge, lasso and elastic net modeling over the classical statistical methods is that, in addition to fitting optimized models, a penalty is applied to predictors in the model, causing covariates with little impact on the outcome variable to be minimized or dropped from the final model. This reduces the model's complexity while increasing its generalizability.

*Logistic regression (LR)* LR is a widely applied statistical model for classification problems. This model applies the maximum likelihood estimation procedure to estimate the parameter of interest. Let $y_{i}$ be the response variable for the ith child, and it is Bernoulli distributed and takes on the value 1 with a probability of $\pi_{i} = P(y_{i} = 1|{\varvec{x}}_{i} )$, where ${\varvec{x}}_{i} = \left( {x_{1} , . . . , x_{p} } \right)^{T}$ is the ith child’s covariate vector, and value 0 with probability 1 − $\pi_{i}$. Then the logistic regression model with the logit link function can be given as:

1 $$\pi_{i} = \frac{{{\text{exp}}\left( {\beta_{0} + {\varvec{x}}_{i}^{T} {\varvec{\beta}}} \right)}}{{1 + {\text{exp}}\left( {\beta_{0} + {\varvec{x}}_{i}^{T} {\varvec{\beta}}} \right)}}$$

where $\beta_{0}$ is the intercept term, and ${\varvec{\beta}} = \left( {\beta_{1} , . . . , \beta_{p} } \right)^{T}$ is a p × 1 vector of estimated regression parameters on the logit scale. If parameter ${\varvec{\theta}} = \left( {\beta_{0} ,{\varvec{\beta}}} \right)^{T}$, then the corresponding log-likelihood function is given by the following equation as it was also shown by [55]:

2 $$l_{{\varvec{\theta}}} = \mathop \sum \limits_{i = 1}^{n} \left[ {y_{i} \log \left( {\pi_{i} } \right) + \left( {1 - y_{i} } \right)\log \left( {1 - \pi_{i} } \right)} \right]$$

By replacing $\pi_{i}$ from Eq. 1 in Eq. 2, we have:

3 $$l_{{\varvec{\theta}}} = \mathop \sum \limits_{i = 1}^{n} \left[ {y_{i} \left( {\beta_{0} + {\varvec{x}}_{i}^{T} {\varvec{\beta}}} \right) - \log \left( {1 + {\text{exp}}(\beta_{0} + {\varvec{x}}_{i}^{T} {\varvec{\beta}}} \right))} \right]$$

In the maximum likelihood method, the goal is finding a set of ${\varvec{\theta}}$ that can maximize Eq. (3). When we have a large number of features (dimensionality), the traditional LR has a few problems: over-fitting, multicollinearity, and computational difficulties. To address this problem, we used regularization which is a GLM that imposes a penalty on the parameters to shrink towards zero [27, 55–58, 63].

*The ridge regression* (L2 regularization, which shrinks coefficients of correlated covariates towards each other) is obtained by maximizing the function with a penalized parameter $\lambda$ applied for all the parameters except the constant (intercept) [55, 56]. The penalized likelihood formulation for ridge regression is given by (4)

4 $$l_{\lambda }^{{\text{R}}} \left( {\varvec{\beta}} \right) = \mathop \sum \limits_{i = 1}^{n} \left[ {y_{i} \left( {{\varvec{x}}_{i}^{T} {\varvec{\beta}}} \right) - \log \left( {1 + {\text{exp}}({\varvec{x}}_{i}^{T} {\varvec{\beta}}} \right))} \right] - \lambda \mathop \sum \limits_{j = 1}^{p} {\varvec{\beta}}_{j}^{2}$$

When the λ values are too large (λ → ∞), the coefficients of all the parameters tend to be zero, but when λ = 0, the ridge regression is equal to the traditional approach.

*The LASSO regression* uses the L-1 penalty for variable selection and shrinkage. As such, if the $\lambda$ is large enough, it forces the coefficient to be zero which provides a lesser number of predictors [57]. The function for the lasso regression is given by (5)

5 $$l_{\lambda }^{{\text{L}}} \left( {\varvec{\beta}} \right) = \mathop \sum \limits_{i = 1}^{n} \left[ {y_{i} \left( {{\varvec{x}}_{i}^{T} {\varvec{\beta}}} \right) - \log \left( {1 + {\text{exp}}({\varvec{x}}_{i}^{T} {\varvec{\beta}}} \right))} \right] - \lambda \mathop \sum \limits_{j = 1}^{p} \left| {{\varvec{\beta}}_{j} } \right|$$

The term $\lambda$ allows the lasso model to carry out much iteration for a given function and find the optimum values for all coefficients. The optimal regularization parameter ($\lambda$) was determined using the nfold cross-validation techniques. The smaller the $\lambda$ value, the more the effect of regularization upon the number of covariates (features) in the model and their respective coefficients [26–28]. Thus, variables with non-zero estimates are considered the important covariates for the outcome variable of interest.

*The elastic net regularization* is a combination of both (3) and (4) penalties [27, 58]. This method can effectively control the group of correlated features and also shrink the coefficients of non-informative features to zero [27, 58, 63, 64]. The elastic net regression is given by (5)

5 $$l_{\alpha }^{{{\text{El}}}} \left( {\varvec{\beta}} \right) = \mathop \sum \limits_{i = 1}^{n} \left[ {y_{i} \left( {{\varvec{x}}_{i}^{T} {\varvec{\beta}}} \right) - \log \left( {1 + {\text{exp}}({\varvec{x}}_{i}^{T} {\varvec{\beta}}} \right))} \right] + \alpha \mathop \sum \limits_{j = 1}^{p} {\varvec{\beta}}_{j}^{2} + \left( {1 - \alpha } \right)\mathop \sum \limits_{j = 1}^{p} \left| {{\varvec{\beta}}_{j} } \right|$$

All the ML algorithms including the logistic regression were performed with R statistical software R and the packages glmnet, pROC, caret, random forest, ggplot, and ROCit were included in the analysis [65–69]. In this paper, we trained the generalized linear model (GLM) estimators with common $\alpha$ values from the set $\left\{ {0,0.5,1} \right\}$, where ($\alpha$ = 0.0, 0.5 and 1.0 respectively refers to the ridge, elastic net and lasso penalty) [27, 58, 63].

*The Random forest (RF)* is the popular supervised ML approach in applied statistics because of its applicability in both classification and regression [70–72]. It is also used for variable screening for dimension reduction. It is a “tree-based” technique in which several decision trees are constructed from a random set of covariates and used to predict an outcome label for a subset of samples. It builds multiple trees (called the forest) and the decision is based on the majority votes over all the trees in the forest [70–73].

*The Neural Network (NN)* is a type of ML algorithm that is made up of layers of nodes, the most important of which are an input layer [74], hidden layers, and output layers. It is set up with several input neurons (X) that represent the information extracted from each feature in the dataset. Back-propagation is a process used in recurrent NN in which prediction errors are fed back through the NN before modifying the weights of each neural connection until the error level is minimized [59, 60].

$$y = activation\left( {\sum (weight + input} \right) + bias)$$

### Model evaluation

**Fig. 2:**
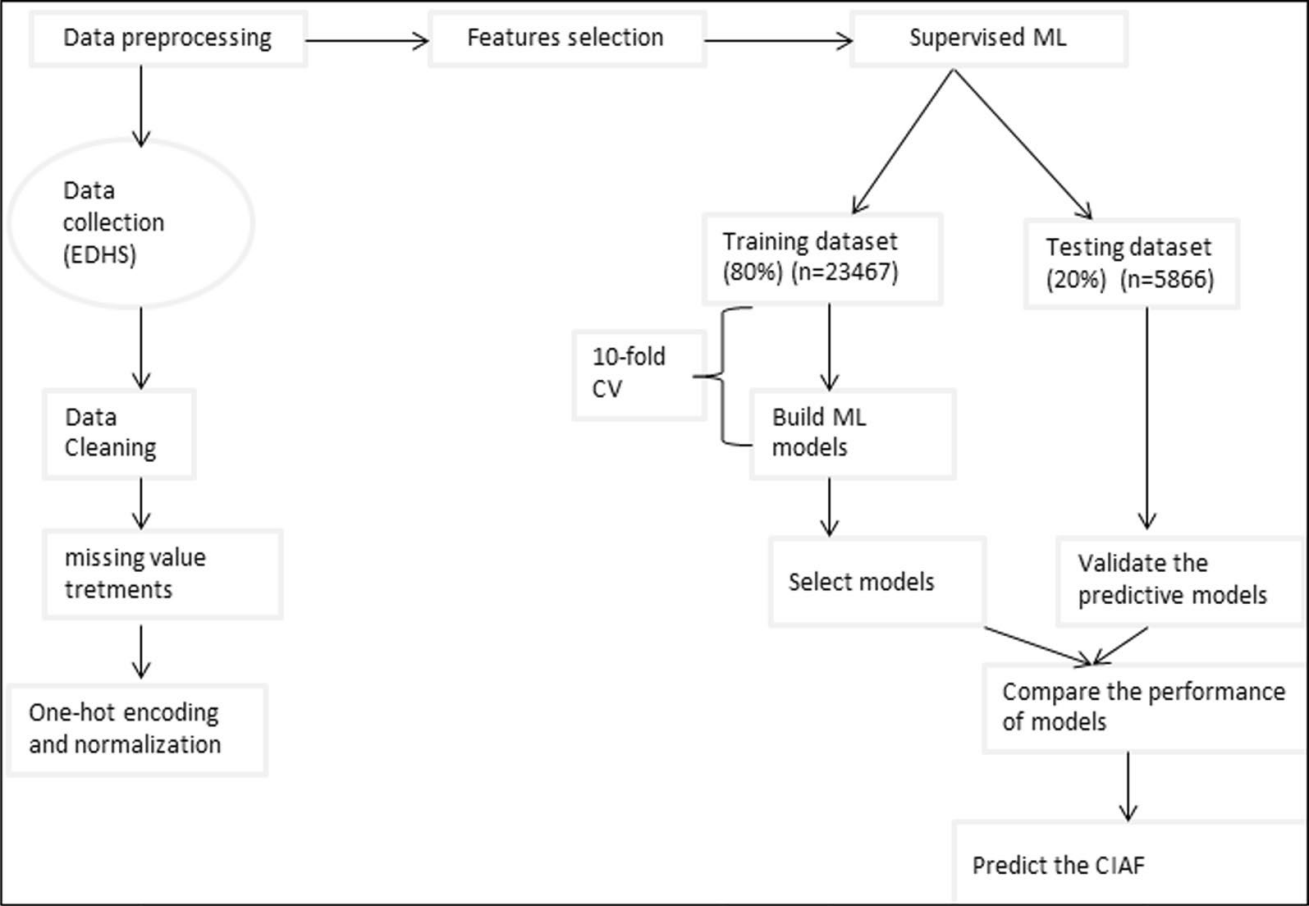
Overview flow chart of the methodologies used

*Model performance* The performances of the given ML models are evaluated using different model performance approaches including sensitivity, specificity, and accuracy [24–29, 75] which are calculated using the observed data as the gold standard. The model sensitivity and specificity relationship are expressed using the Receiver operating characteristics (ROC) curves (Fig. 2).

All the curves which are plotted to the left of the diagonal line are performing better than chance. The area under each curve (AUC) gives an aggregated value which explains the probability that a random sample would be correctly classified by each of the ML algorithms [25, 76]. The AUC of the ROC curve averaged over 10 cross-validation folds (ten repeats) [25], which partitions the original sample into ten disjoint subsets, uses nine of those subsets in the training process, and then makes predictions about the remaining subset. Then the identified best-fit model is used to predict the undernutrition in another dataset, known as the test dataset [24–29].

*Covariate selection and ranking* Covariate selection is very important for prediction and interpretations, especially for high-dimensional datasets. To assess the importance of predictors in the selected model, the study employed two important measures; Mean Decreases Accuracy (MDA) and Mean Decrease Gini (MDG). The highest decrease in the accuracy and Gini values of the model implies the best predictive and the most important variable respectively [77] for the successful classifications (Table 1).

**Table 1:** The description of the response variable and the respective covariates included in the model

	**Descriptions**
Childhood undernutrition using CIAF (outcome variable)	$$yi = \left\{ {\begin{array}{*{20}l} {1:} \hfill & {if \,a \,child\, i \,had\, at\, least\, one\, form\, of\, undernutrion\, \left( { CIAF} \right) } \hfill \\ {0:} \hfill & { if \,child \,i\, is\, nourished } \hfill \\ \end{array} } \right.$$
*Children level covariates*	
Sex	Sex of a child (female vs male)
Age (months)	Age of a child in months
Vitamin A (VA)	Yes/no
Birth order (BO)	1, 2–3 4 + (birth order number)
Breastfeeding (BF)	Yes/no
Child comorbidity status (CO)	(Presence of diarrhea, fever, ALRI in last 2 weeks before the survey): (No vs yes)
Types of birth (TB)	(Multiple vs singleton)
Size of the child at birth (SC)	(Smaller than average, average, larger than average)
Dietary diversity score (DDS)	Below minimum requirement/ minimum requirement
*Maternal/household-level covariates*	
Mother’s age	15–24, 25–34 and 35–49 (respondents current age 15–49)
Residence (PR)	Rural/urban
Mother’s educational level (ME)	No formal education, primary and secondary and above
Father’s educational level (FE)	No formal education, primary and secondary and above
Women’s autonomy tertiles (WA)	Low, medium, and high
Toilet facility (TF)	Improved and unimproved
Source of drinking water (SDW)	Improved and unimproved
BMI	Body mass index of mothers (< 18.5 kg/m^2^, 18.5–24.9 kg/m^2^ and ≥ 25 kg/m^2^)
Number of children under five (NUFC)	Number of children under the age of 5 (0–1, 2, 3 or more)
Survey year (SY)	Years of the survey (2000, 2005, 2011, and 2016)
Media exposure	Yes/no
Working status of the mother (WS)	Not working/working
Household size	< 4, 5–9, 10 + (continuous)
Wealth quantile (WQ)	Poorest, poor, middle, richer, and richest
*Geospatial covariates*	
Precipitation (precp)	The average precipitation measured within the 10 km (rural) or 2 km (urban)
Aridity index	The ratio of annual precipitation to annual potential evapotranspiration (10 km × 10 km)
Evaporation	Global elevation above earth’s sea level
Maximum temperature (MaxT)	The average annual maximum temperature within the 10 km (rural) or the 2 km (urban)
Minimum temperature (MinT)	The average annual minimum temperature within the 10 km (rural) or the 2 km (urban)
Potential evaporation (pet)	The average annual pet within the 10 km (rural) or the 2 km (urban)
Proximity to water (proxtmty)	Straight-line distance to the nearest major water body
Urban–rural settlement (UR)	This is the urban–rural population classification of the area within the 10 km (rural) or the 2 km (urban)
Population density (popD)	Estimates of human population density is the number of persons/km^2^
Enhanced vegetation index (EVI)	The average vegetation index value within the 10 km (rural) or the 2 km (urban)
Cluster altitude (Alt)	Cluster altitude
Wet days (WetD)	The average number of days receiving rainfall within the 10 km (rural) or 2 km (urban)

## Results

This analysis consisted of data from 29,333 children of age 0–59 months. Of these, 15,281 (52.09%) had at least one form of the undernutrition indicators (stunting, wasting, and underweight) measured in terms of CIAF. We examined the prevalence of CIAF of U5C experience across different child and mother-household level covariates. The prevalence of CIAF was more common among parents with no formal education compared to parents with secondary and post-secondary levels of educations. Most of the undernourished children were from rural areas. Also, the prevalence of undernourished children was reported from the lower wealth index of households, from mothers having no media exposure, from unimproved toilets and sanitation compared with their counterparts. Covariates that were significant in the Chi-square statistics were used to develop the ML algorithms on the training dataset (Table 2).

**Table 2:** Sample characteristics (n = 29,333)

Variables	Categories	Nourished (%)	CIAF (%)	X^2^ test statistic	*p* values
Sex of child	Male	44.74	55.26	29.96	< 0.001
	Female	47.93	52.07		
Age of a child (months)	< 23	55.40	44.60	652.83	< 0.001
	24–59	40.25	59.78		
Vitamin A	Yes	43.75	56.25	75.02	< 0.001
	No	48.79	51.21		
Birth order	1	50.36	49.64	67.89	< 0.001
	2–3	47.55	52.45		
	4 +	44.17	55.83		
Breastfeeding	Yes	46.21	53.79	0.14	0.707
	No	46.59	53.41		
Comorbidity	Yes	43.17	56.83	58.25	< 0.001
	No	47.88	52.12		
Size of the child at birth	Smaller than average	46.30	53.7	268.357	< 0.001
	Average	48.06	51.94		
	Larger than average	50.9	49.10		
Dietary diversity score (DDS)	Below minimum	46.56	53.44	2.432	0.349
	Minimum	45.51	54.49		
Types of birth	Singleton	46.62	53.38		
	Multiple	29.17	70.83		
Mother’s age	15–24	48.51	51.49	31.06	< 0.001
	25–34	46.46	53.54		
	35–49	43.91	56.09		
Place of residence	Rural	44.61	55.39	285.50	< 0.001
	Urban	60.58	39.42		
Mother’s education	No formal education	42.98	57.02	510.57	< 0.001
	Primary	51.81	48.19		
	Secondary and above	69.85	30.15		
Father’s education	No formal education	41.09	58.91	475.61	< 0.001
	Primary	49.68	50.32		
	Secondary and above	59.81	40.19		
Woman’s autonomy tertiles	Low autonomy	44.11	55.89	49.84	< 0.001
	Middle autonomy	47.60	52.40		
	High autonomy	48.84	51.16		
Source of drinking water	Unimproved	44.77	55.23	20.04	0.009
	Improved	47.41	52.59		
Toilet facilities	Unimproved	41.29	58.71	442.18	< 0.001
	Improved	53.77	46.23		
BMI	Underweight	40.46	59.54	278.85	< 0.001
	Normal	46.85	53.15		
	Overweight	66.01	33.99		
Household number	Less than 4	48.42	51.58	18.74	0.017
	5–9	45.48	54.52		
	$\ge \hspace{0.17em}$10	47.22	52.78		
Number of under-five children in HH	1	47.19	52.81	62.44	< 0.001
	2	44.23	55.77		
	3 or more	50.43	49.57		
Media exposure	No	43.03	56.97	205.71	< 0.001
	Yes	51.63	48.37		
Mother’s working status	Unemployed	48.14	51.86	65.67	< 0.001
	Employed	43.27	56.73		
Wealth quintile	Poorest	40.31	59.69	343.16	< 0.001
	Poorer	42.56	57.44		
	Middle	45.92	54.05		
	Richer	48.56	51.44		
	Richest	56.67	43.33		
EDHS	2000	38.81	61.19	394.42	< 0.001
	2005	43.62	56.38		
	2011	48.54	51.46		
	2016	53.34	46.66		

Figures in the supplementary documents indicated the effects of different levels of the log of the regularization parameter ($\lambda$) for the ridge, elastic net, and lasso regression using the dotted vertical lines (here at x = − 4.51, x = − 7.84, and x = − 8.71) respectively, which indicates the accuracy of the prediction maximization. The coefficients for the given model features were indicated for different values of log ($\lambda$) that minimizes a mean squared error (MSE) of coefficients established during the cross-validation. The graph shows that as the log ($\lambda$) value decreases, the number of the variables included in the model (those with nonzero coefficients) increases (Additional file 1).

**Fig. 3:**
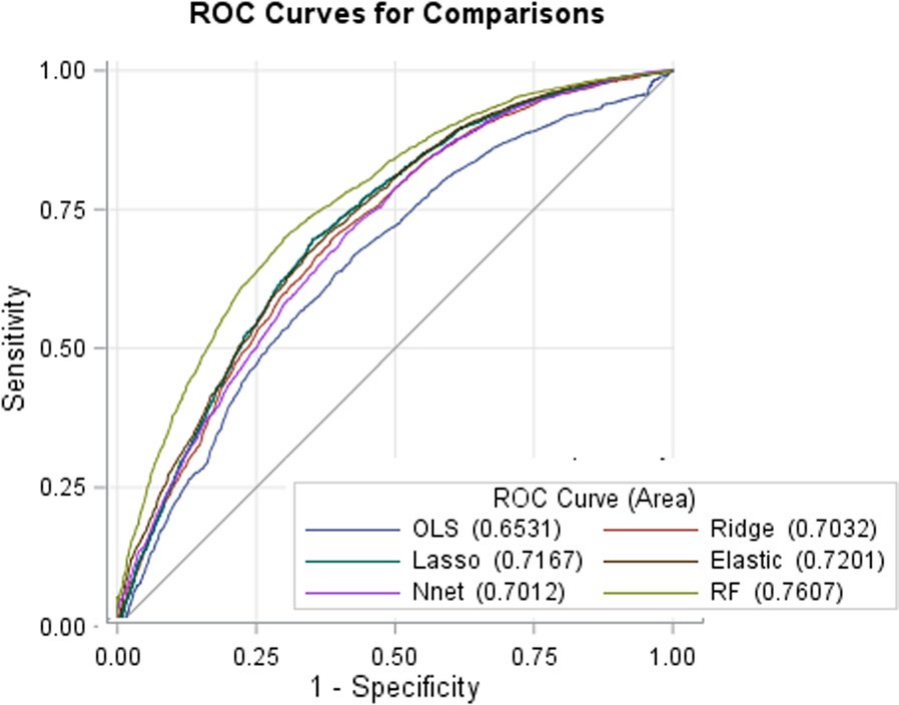
ROC curves for LR, L-1 regularization, L-2 regularization, elastic net regularization, ANN, and RF in predicting undernutrition among under-five children

*Performance comparisons* The accuracy and AUC were implemented to evaluate the efficiency of ML algorithms. The comparison of the efficiency of ML algorithms with the traditional LR was depicted in Fig. 3 and Table 3. All the ML algorithms considered in this study perform better than those of the classical logistic regression model to predict the undernutrition status. More detail is given in the Additional file 1.

**Table 3:** The performance of the prediction models based on different classifications on the independent tests for two ratios

Train/test ratios	Algorithms	Sensitivity	Specificity	Precision	F1	AUC (95% CI)	Accuracy (95% CI)
80/20	GLM	0.585	0.169	0.399	0.475	0.630 (0.619, 0.641)	0.371 (0.359, 0.383)
	Ridge	0.503	0.789	0.683	0.580	0.699 (0.686, 0.713)	0.645 (0.633, 0.658)
	Lasso	0.484	0.814	0.711	0.576	0.711 (0.698, 0.724)	0.654 (0.641, 0.666)
	elastic-net	0.484	0.802	0.697	0.572	0.701 (0.689, 0.714)	0.647 (0.635, 0.660)
	NN	0.499	0.785	0.686	578	0.697 (0.684, 0.711)	0.646 (0.634, 0.658)
	RF	0.524	0.819	0.732	0.611	0.756 (0.744, 0.769)	0.676 (0.663, 0.688)
70/30	GLM	0.601	0.189	0.361	0.445	0.653 (0.639, 0.667)	0.356 (0.344, 0.369)
	Ridge	0.510	0.804	0.743	0.604	0.703 (0.690, 0.717)	0.649 (0.636, 0.661)
	Lasso	0.516	0.819	0.698	0.593	0.717 (0.704, 0.730)	0.683 (0.671, 0.695)
	Elastic-net	0.527	0.824	0.717	0.608	0.720 (0.707, 0.733)	0.682 (0.670, 0.694)
	NN	0.499	0.785	0.751	0.621	0.701 (0.688, 0.715)	0.656 (0.644, 0.668)
	RF	0.524	0.819	0.715	0.595	0.761 (0.749, 0.773)	0.688 (0.676, 0.700)

A comparison of 70% training and 30% validation, 80% training and 20% validation was performed respectively to examine the six models’ behaviors with some statistical measures and area under the receiver operating characteristic curve. Although all the models with the two train-test splits ratio had almost identical performances evaluation metrics, the 70–30% split was chosen as the most appropriate model to undernutrition classification. Moreover, it was noticed that the prediction model based on RF demonstrated the best-performed model, with AUC up to 0.761, followed by LASSO (AUC = 0.717), while the perdition model using the traditional model (LR) is the least efficient (AUC = 0.653). Hence RF model was chosen as the classification engine to construct the perdition model for under-five undernutrition in Ethiopian administrative zones (Table 3).

**Fig. 4:**
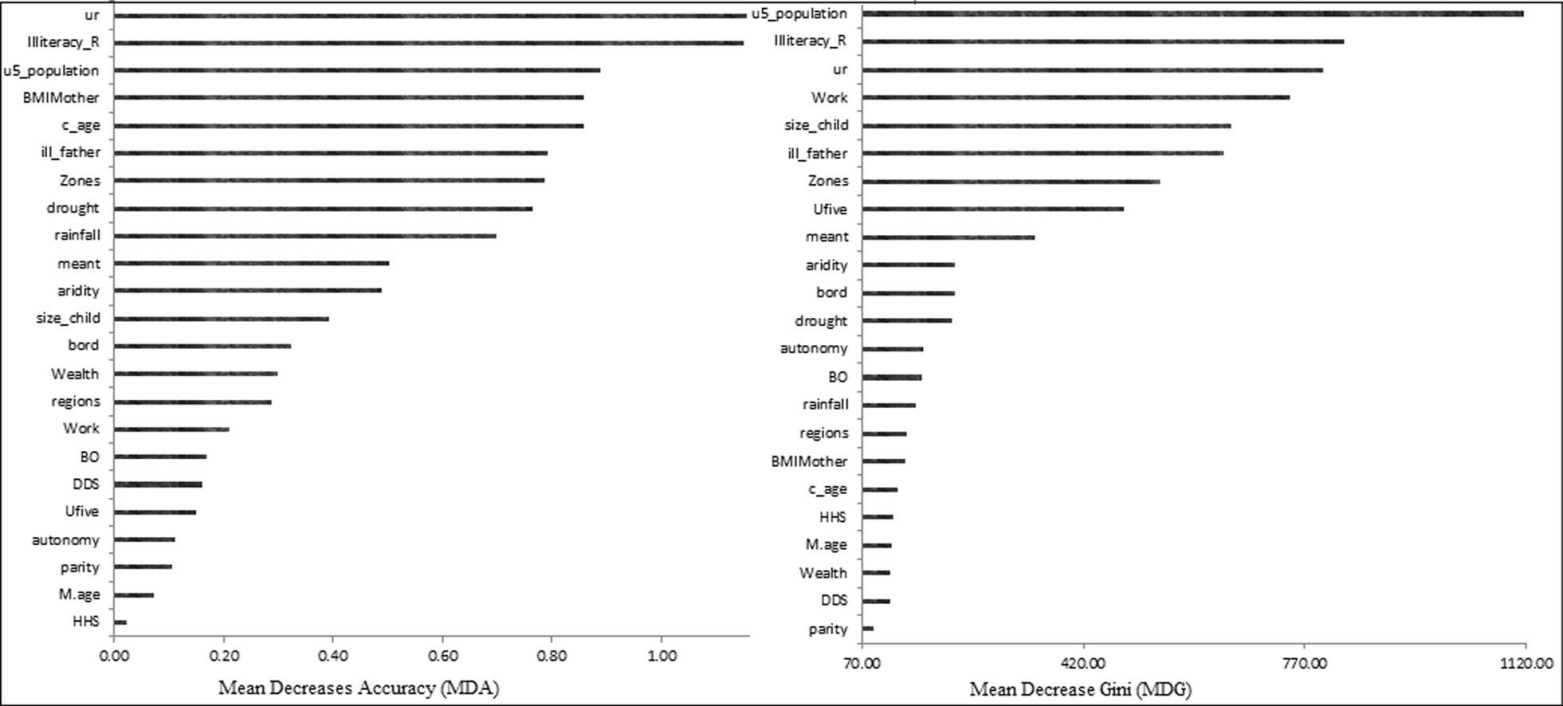
Relative Variable importance from the best model (random forest)

In machine learning prediction, identifying important attributes is also crucial. The importance of each aspect for a tree’s decision is represented by feature importance rates. The random forest (best algorithm for childhood undernutrition in our study gives the MDA and MDG measures of the relative importance of covariates in the model which are summarized in Fig. 4. The factors include urban–rural settlement (ur), the total number of under-five population, the BMI, literacy rates of parents and zones were the most important predictors of CIAF, but household size, age of mother, parity, and autonomy were the lowest predictive variables in our model (Fig. 4).

**Fig. 5:**
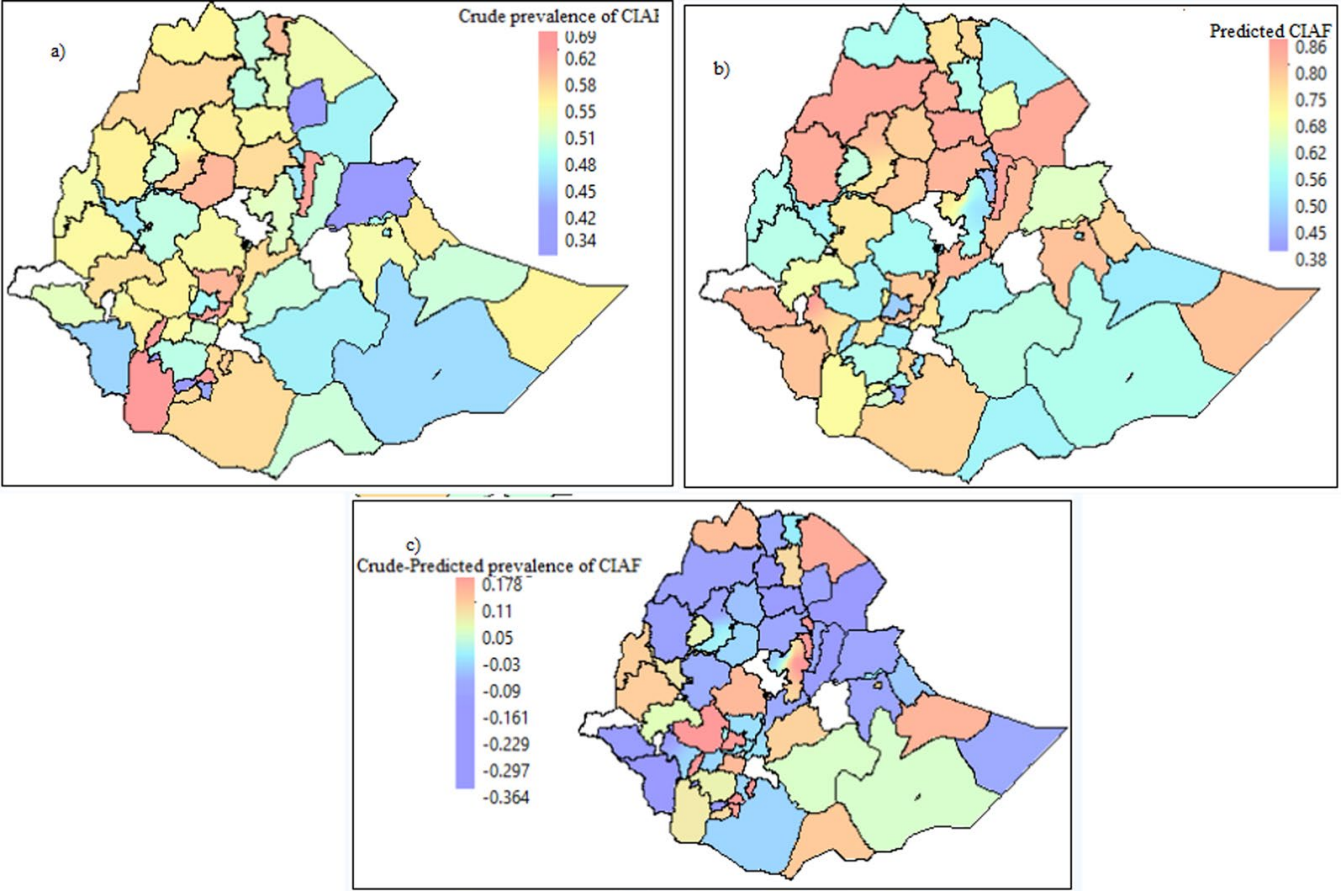
mapping the predicted and actual prevalence of undernutrition outcomes based on the test data

The predicted values with the actual values of undernutrition among the 72 administrative areas were mapped in Fig. 5. Having the best predictive model (RF) that yielded the highest AUC, we further predicted the undernutrition status of under-five children by the administrative zones. Both the crude and predicted undernutrition values were merged with the second-level administrative level (zones) shapefiles. A visual comparison confirms that while discrepancies did exist between few zones, the overall patterns of the observed prevalence were in line with the patterns of the predicted prevalence of undernutrition. The degrees of agreement between the actual and predicted values indicated that the two variables are strongly correlated. Moreover, the third map reveals that the difference. Further, it is between the crude and predicted CIAF of U5C in some zones that have a positive difference indicated that the crude prevalence is less than the predicted value and vice versa (Fig. 5).

## Discussions

Previous studies carried out on this subject reported that Ethiopia is one of the countries with the highest number of under-five undernourished children in the world [2, 4, 8, 78, 79]. Further, the studies indicated that, while the prevalence of under-five undernutrition has declined in the nation from time to time, more effort is needed to facilitate this decline and to contain the negative consequences of the phenomena. In this study, we briefly described spatial disparities in under-five undernutrition and predicted under-five undernutrition among Ethiopian administrative zones. The spatial maps show evidences of considerable zonal disparities in under-five undernutrition rates in the administrative zones similar to what has been reported in different countries [80–82]. The continuous data in this study were normalized and the categorical variables were encoded. The machine learning models are known as advanced approaches and techniques for quick and accurate prediction of real-world problems. In this paper, the ML techniques are analyzed by investigating the influence of training/testing ratio on the performance of the six popular ML models to predict the undernutrition of under-five children. The performance of the ML models was slightly changed under the two different ratios. The result revealed that the ratio 70/30 was the most suitable ratio for the training and validating ML models. This study is in line with previously published studies [18, 23, 30–44, 83–86]. The ML tool can offer insight into the identification of novel factors associated with under-five undernutrition that can serve as targets for intervention. Among the six predictive models built using these techniques, the Random Forest (RF) model reveals a higher predictive power as compared to other ML models including the logistic regression. The RF model reveals that urban–rural settlement ratio, the literacy level of parents, under five populations, BMI of mothers, locations (zones, place of residence), and rainfall distributions were the top important predictors of under-five undernutrition in Ethiopia. This study is consistent with previous studies [4, 42, 79, 81]. Moreover, the selected ML algorithm reveals consistent effects of the covariates with the classical generalized linear model which shows that the educational level of parents, the age of the child, sex of the child, birth order, dietary diversity, types of the birthplace of residence, women’s autonomy, household sanitation, and a clean water supply were the most significant variables for undernutrition [4, 6, 7, 10, 21, 79–82]. The child’s residence (zones) was one of the important risk factors for the U5C CIAF rate which varied significantly across spatial zones. Moreover, this paper briefly explored the spatial variation in under-five child undernutrition and the predicted under-five undernutrition risk factors in Ethiopia using the different machine learning approaches. Hence, we explored a spatial map for the crude prevalence and predicted (from RF) rate of under-five undernutrition by zones in Ethiopia to document the zonal disparities in under-five undernutrition in the country.

## Limitations

Since there are no regression coefficients and no directional effects in ML algorithms, the parameters are difficult to be interpreted [21, 23, 87]. In the current study, ML models only predict or classify certain variables depending on the importance of their contribution in determining under-five undernutrition instead of causal inferences. More types of classification ML algorithms could also have been used [21, 23, 28, 38, 59].

## Conclusions

The main objective of this study was to compare and evaluate the performance of different machine learning (ML) algorithms considering the influence of two train-test splits ratios in predicting the undernutrition under-five classification. Popular statistical indicators, such as accuracy and area under the curve were employed to evaluate the predictive power of the ML models under different testing and training ratios. The higher the accuracy the model had, the better was the performance of the model. Our results confirm that ML models can effectively predict the under-five undernutrition status and hence may be useful for concerned body decision tools. The best model was the RF, with accuracy and AUC of (68.2%, 76.2%) respectively. The findings from this paper showed that considerable zonal disparities in the under-five undernutrition status persist in the northern part of Ethiopia. When implementing health policies aimed at the redaction of child undernutrition in Ethiopian administrative zones, the zone characteristics must be taken into account.

## Supplementary Information

**Additional file 1:** Implementation of different Supervised Machine Learning (SML) using R statistical software.

## Data Availability

The dataset used and analyzed during the current study is available from the corresponding author on reasonable request.

## Abbreviations

AUC: Area under the curve
EDHS: Ethiopian Demographic and Health Survey
BMI: Body mass index
ML: Machine learning
RF: Random forest
ANN: Artificial neural network
ROC: Receiver operating characteristic
U5C: Under-five children
LR: Logistic regression
