# Supplementary material for: A machine learning classifier approach for identifying the determinants of under-five child undernutrition in Ethiopian administrative zones
Source: BMC Med Inform Decis Mak. 2021 Oct 24;21:291. doi: 10.1186/s12911-021-01652-1 (PMC8542294; doi:10.1186/s12911-021-01652-1)
Supplement: Supplementary file 1 — Additional file 1: Implementation of different Supervised Machine Learning (SML) using R statistical software. [file 12911_2021_1652_MOESM1_ESM.html]

Machine Learning Algorithms


# Machine Learning Algorithms

#### Haile Mekonnen

#### 7/15/2021

```
library(pROC)
```

```
## Type 'citation("pROC")' for a citation.
```

```
## 
## Attaching package: 'pROC'
```

```
## The following objects are masked from 'package:stats':
## 
##     cov, smooth, var
```

```
library(caret)
```

```
## Loading required package: lattice
```

```
## Loading required package: ggplot2
```

```
setwd("D:/PhDnurtion/papers/paper 5")
library(readxl)#used to import the excel file
CIAF <- read_excel("finaldata.xls")
finaldata <- read_excel("finaldata.xls")
CIAF = as.data.frame(CIAF)
#View(CIAF)###we can see the spreadsheet of the dataset
```

```
#CIAF <- CIAF[ which(CIAF$dhs_year==4), ]
############missing value managements in the dataset
#1. identify if there is any missing values 
# 2. remove if not much
#################################### 
#is.na(CIAF$zpopulation)#toc check weather there is a missing value or not
# Recode missing values with NA.
# Replace NA by the average for the continuous variable 
CIAF$zaridity1[is.na(CIAF$zaridity1)]=mean(CIAF$zaridity1,na.rm=TRUE)
CIAF$zpopulation[is.na(CIAF$zpopulation)]=mean(CIAF$zpopulation,na.rm=TRUE)
CIAF$zpreciptitation[is.na(CIAF$zpreciptitation)]=mean(CIAF$zpreciptitation,na.rm=TRUE)
CIAF$zur[is.na(CIAF$zur)]=mean(CIAF$zur,na.rm=TRUE)
CIAF$zdrought[is.na(CIAF$zdrought)]=mean(CIAF$zdrought,na.rm=TRUE)
CIAF$zevi[is.na(CIAF$zevi)]=mean(CIAF$zevi,na.rm=TRUE)
CIAF$zirrigation[is.na(CIAF$zirrigation)]=mean(CIAF$zirrigation,na.rm=TRUE)
CIAF$zlst[is.na(CIAF$zlst)]=mean(CIAF$zlst,na.rm=TRUE)
CIAF$zmaxt[is.na(CIAF$zmaxt)]=mean(CIAF$zmaxt,na.rm=TRUE)
CIAF$zmint[is.na(CIAF$zmint)]=mean(CIAF$zmint,na.rm=TRUE)
CIAF$zpet[is.na(CIAF$zpet)]=mean(CIAF$zpet,na.rm=TRUE)
CIAF$zelevition[is.na(CIAF$zelevition)]=mean(CIAF$zelevition,na.rm=TRUE)
CIAF$zwetd[is.na(CIAF$zwetd)]=mean(CIAF$zwetd,na.rm=TRUE)
CIAF$zu5_population[is.na(CIAF$zu5_population)]=mean(CIAF$zu5_population,na.rm=TRUE)
########
# Recode the CIAF (outcome) variable to 0 and 1.
#tail(CIAF,10) # Show the last 10 rows of the dataset in the list
# To improve the reproducibility of our analysis, we set the seed of the random generator 
set.seed(123456) # Set a random seed so that repeated analyses have the same outcome.
##Seeds are saved on the PC only and will not allow analyses to be repeated precisely on other machines.
index = 1:nrow(CIAF) #Create an index vector with as many sequential 
#variables as there are rows in the CIAF dataset.
testindex = sample(index, trunc(length(index)/5)) #Take a sample of 20% of the observations from 
#the index vector.
testset = CIAF[testindex, ] #Create a test (validation) dataset with 20% of the CIAF dataset.
trainset = CIAF[-testindex, ] #Create a trainig dataset with 80% of the data.
```

```
x_train = data.matrix(trainset[,c(6,7,10:73)]) # Take the features (x) from the training dataset.
y_train = as.numeric(trainset[, 74]) # Take the outcomes (y) from the training dataset.
x_test = data.matrix(testset[,c(6,7,10:73)]) # Take the features (x) from the testing/validation dataset.
y_test = as.numeric(testset[, 74]) # Take the outcomes (y) from the testing/validation dataset.
# You can use the dim() function to assess the dimension of each matrix
```

```
# (e.g., dim(x_train))
# install.packages('glmnet',repos=getOption('repos')) Install latest verison
# of `glmnet`. Only necessary once.
################################################
```

##OLS

```
################################################
attach(CIAF)
library(glmnet)
```

```
## Loading required package: Matrix
```

```
## Loaded glmnet 4.1-1
```

```
###ordinal glm (binry logistic regression)
```

```
ols.model = cv.glmnet(x_train, y_train, alpha=0, nfolds=10) # 10-fold cross validation of the LASSO-regulated linear model.
lambda.min = ols.model$lambda.min # Save the lambda value which minimizes the error of the linear model.
glm_coef = round(coef(ols.model,s= 0),2) #Individual coefficients for variable included in the model.
plot(ols.model) # Plots mean squared error against log(Lambda).
```

```
plot(glmnet(x_train,y_train, family="gaussian", alpha=0),"lambda",label=T, main="") #Plots coefficient values againt log(Lambda)
abline(v=log(lambda.min), lty=3) #Adds a vertical line to the plot of line 34 at the minimum level of l
```

```
log(lambda.min)
```

```
## [1] -4.318666
```

##Ridge regression

```
ridge_model = cv.glmnet(x_train, y_train, alpha=0, nfolds=10) # 10-fold cross validation of the LASSO-regulated linear model.
lambda.min = ridge_model$lambda.min # Save the lambda value which minimizes the error of the linear model.
glm_coef = round(coef(ridge_model,s= lambda.min),2) #Individual coefficients for variable included in the model.
plot(ridge_model) # Plots mean squared error against log(Lambda).
```

```
plot(glmnet(x_train,y_train, family="gaussian", alpha=0),"lambda",label=T, main="") #Plots coefficient values againt log(Lambda)
abline(v=log(lambda.min), lty=3) #Adds a vertical line to the plot of line 34 at the minimum level of l
```

```
log(lambda.min)
```

```
## [1] -4.504733
```

# 

##Lasso regression

```
Lasso_model = cv.glmnet(x_train, y_train, alpha=1, nfolds=10) # 10-fold cross validation of the LASSO-regulated linear model.
lambda.min = Lasso_model$lambda.min # Save the lambda value which minimizes the error of the linear model.
glm_coef = round(coef(Lasso_model,s= lambda.min),2) #Individual coefficients for variable included in the model.
plot(Lasso_model) # Plots mean squared error against log(Lambda).
```

```
plot(glmnet(x_train,y_train, family="gaussian", alpha=1),"lambda",label=T, main="") #Plots coefficient values againt log(Lambda)
abline(v=log(lambda.min), lty=3) #Adds a vertical line to the plot of line 34 at the minimum level of l
```

```
log(lambda.min)
```

```
## [1] -8.71451
```

##Elastic Net regression

```
elastic_model = cv.glmnet(x_train, y_train, alpha=0.5, nfolds=10) # 10-fold cross validation of the LASSO-regulated linear model.
lambda.min = elastic_model$lambda.min # Save the lambda value which minimizes the error of the linear model.
glm_coef = round(coef(elastic_model,s= lambda.min),2) #Individual coefficients for variable included in the model.
plot(elastic_model) # Plots mean squared error against log(Lambda).
```

```
plot(glmnet(x_train,y_train, family="gaussian", alpha=1),"lambda",label=T, main="") #Plots coefficient values againt log(Lambda)
abline(v=log(lambda.min), lty=3) #Adds a vertical line to the plot of line 34 at the minimum level of l
```

```
log(lambda.min)
```

```
## [1] -7.835295
```

```
#
# Create a vector of predictions made from the test/validation data set for OLS, Ridge, Lasso and Elastic.
ols_pred = round(predict(ols.model, x_test, type="response"),3) 
ridge_pred = round(predict(ridge_model, x_test, type="response"),3)
Lasso_pred = round(predict(Lasso_model,x_test, type="response"),3) 
Elastic_pred = round(predict(elastic_model,x_test, type="response"),3)
```

```
require(nnet) # Load e1071 package into this R session.
```

```
## Loading required package: nnet
```

```
library(nnet)
## Loading required package: nnet
nnet_model = nnet(x_train, y_train, size=5) #Fit a single-layer neural network to the data with 5 units in the hidden layer.
```

```
## # weights:  341
## initial  value 6298.936357 
## iter  10 value 5599.590717
## iter  20 value 5338.083270
## iter  30 value 5223.247933
## iter  40 value 5197.695576
## iter  50 value 5162.223415
## iter  60 value 5138.344666
## iter  70 value 5125.601365
## iter  80 value 5118.571866
## iter  90 value 5101.374015
## iter 100 value 5089.557056
## final  value 5089.557056 
## stopped after 100 iterations
```

```
###Prediction vector for the neural network.
nnet_pred = round(predict(nnet_model, x_test, type="raw"),3) 
#install.packages("rpart.plot")
library(rpart)
library(rpart.plot)
train_data=data.frame(y_train,x_train)
fit <-rpart(train_data$y_train~.,data = train_data,method = "class")
#rpart.plot(fit, extra = 106)
x_test=data.frame(x_test)
#Prediction vector for the random forest
RF_pred <-round(predict(fit,x_test),3)
# Collect the six prediction vectors into a data frame.
predictions = data.frame(y_test, ols_pred,ridge_pred,Lasso_pred, Elastic_pred,nnet_pred,RF_pred)
names(predictions) = c("CIAF", "ols","ridge","lasso","Elastic","nnet","RF" )
#Name the columns of the dataframe.
head(predictions,10)#list the first 10 predicted observations
```

```
##       CIAF   ols ridge lasso Elastic  nnet    RF    NA
## 19516    1 0.601 0.607 0.618   0.621 0.644 0.391 0.609
## 16618    1 0.329 0.323 0.337   0.328 0.224 0.733 0.267
## 25642    0 0.349 0.350 0.355   0.353 0.227 0.628 0.372
## 22385    1 0.643 0.646 0.620   0.627 0.705 0.391 0.609
## 23678    1 0.355 0.349 0.341   0.339 0.223 0.733 0.267
## 12999    0 0.692 0.698 0.695   0.702 0.653 0.391 0.609
## 2285     0 0.488 0.489 0.495   0.490 0.348 0.391 0.609
## 6326     0 0.588 0.585 0.588   0.580 0.707 0.391 0.609
## 10982    1 0.195 0.192 0.215   0.204 0.244 0.628 0.372
## 19530    1 0.611 0.613 0.643   0.642 0.493 0.391 0.609
```

```
tail(predictions)#list the last six predicted values
```

```
##       CIAF   ols ridge lasso Elastic  nnet    RF    NA
## 17967    1 0.623 0.624 0.621   0.627 0.618 0.391 0.609
## 17041    0 0.525 0.526 0.552   0.550 0.525 0.391 0.609
## 3662     1 0.648 0.653 0.673   0.674 0.656 0.391 0.609
## 23267    1 0.568 0.571 0.565   0.569 0.687 0.628 0.372
## 920      0 0.583 0.583 0.576   0.576 0.478 0.391 0.609
## 14080    1 0.265 0.255 0.262   0.243 0.221 0.733 0.267
```

```
write.csv(predictions, file = "datasas1.csv")#import the data to csv format for further analsis
```

```
library(readr)
Hsas <- read_csv("Rcodes/Hsas.csv")
```

```
## 
## -- Column specification --------------------------------------------------------
## cols(
##   CIAF = col_double(),
##   OLS = col_double(),
##   ridge = col_double(),
##   lasso = col_double(),
##   Elastic = col_double(),
##   nnet = col_double(),
##   RF = col_double()
## )
```

```
ols=round(Hsas$OLS,0)
ridge=round(Hsas$ridge,0)
lasso=round(Hsas$lasso,0)
Elastic=round(Hsas$Elastic,0)
nnet=round(Hsas$nnet,0)
RF=round(Hsas$RF,0)
CIAF=Hsas$CIAF
dataco=data.frame(CIAF,ols,ridge,lasso,Elastic,nnet,RF)
head(dataco)
```

```
##   CIAF ols ridge lasso Elastic nnet RF
## 1    1   0     1     1       1    1  1
## 2    1   0     1     1       1    1  1
## 3    1   0     1     1       1    1  1
## 4    1   0     1     1       1    1  1
## 5    1   0     1     1       1    1  1
## 6    1   0     1     1       1    1  1
```

```
confusionMatrix(as.factor(dataco$ols),as.factor(dataco$CIAF)) # Create a confusion matrix for the SVM.
```

```
## Confusion Matrix and Statistics
## 
##           Reference
## Prediction    0    1
##          0 1658 2495
##          1 1177  507
##                                           
##                Accuracy : 0.3709          
##                  95% CI : (0.3585, 0.3834)
##     No Information Rate : 0.5143          
##     P-Value [Acc > NIR] : 1               
##                                           
##                   Kappa : -0.2431         
##                                           
##  Mcnemar's Test P-Value : <2e-16          
##                                           
##             Sensitivity : 0.5848          
##             Specificity : 0.1689          
##          Pos Pred Value : 0.3992          
##          Neg Pred Value : 0.3011          
##              Prevalence : 0.4857          
##          Detection Rate : 0.2841          
##    Detection Prevalence : 0.7115          
##       Balanced Accuracy : 0.3769          
##                                           
##        'Positive' Class : 0               
##
```

```
confusionMatrix(as.factor(dataco$ridge),as.factor(dataco$CIAF)) # Create a confusion matrix for the SVM.
```

```
## Confusion Matrix and Statistics
## 
##           Reference
## Prediction    0    1
##          0 1427  662
##          1 1408 2340
##                                           
##                Accuracy : 0.6454          
##                  95% CI : (0.6329, 0.6576)
##     No Information Rate : 0.5143          
##     P-Value [Acc > NIR] : < 2.2e-16       
##                                           
##                   Kappa : 0.2849          
##                                           
##  Mcnemar's Test P-Value : < 2.2e-16       
##                                           
##             Sensitivity : 0.5034          
##             Specificity : 0.7795          
##          Pos Pred Value : 0.6831          
##          Neg Pred Value : 0.6243          
##              Prevalence : 0.4857          
##          Detection Rate : 0.2445          
##    Detection Prevalence : 0.3579          
##       Balanced Accuracy : 0.6414          
##                                           
##        'Positive' Class : 0               
##
```

```
confusionMatrix(as.factor(dataco$lasso),as.factor(dataco$CIAF)) # Create a confusion matrix for the SVM.
```

```
## Confusion Matrix and Statistics
## 
##           Reference
## Prediction    0    1
##          0 1373  559
##          1 1462 2443
##                                          
##                Accuracy : 0.6538         
##                  95% CI : (0.6414, 0.666)
##     No Information Rate : 0.5143         
##     P-Value [Acc > NIR] : < 2.2e-16      
##                                          
##                   Kappa : 0.3008         
##                                          
##  Mcnemar's Test P-Value : < 2.2e-16      
##                                          
##             Sensitivity : 0.4843         
##             Specificity : 0.8138         
##          Pos Pred Value : 0.7107         
##          Neg Pred Value : 0.6256         
##              Prevalence : 0.4857         
##          Detection Rate : 0.2352         
##    Detection Prevalence : 0.3310         
##       Balanced Accuracy : 0.6490         
##                                          
##        'Positive' Class : 0              
##
```

```
confusionMatrix(as.factor(dataco$Elastic),as.factor(dataco$CIAF)) # Create a confusion matrix for the SVM.
```

```
## Confusion Matrix and Statistics
## 
##           Reference
## Prediction    0    1
##          0 1373  596
##          1 1462 2406
##                                          
##                Accuracy : 0.6474         
##                  95% CI : (0.635, 0.6597)
##     No Information Rate : 0.5143         
##     P-Value [Acc > NIR] : < 2.2e-16      
##                                          
##                   Kappa : 0.2882         
##                                          
##  Mcnemar's Test P-Value : < 2.2e-16      
##                                          
##             Sensitivity : 0.4843         
##             Specificity : 0.8015         
##          Pos Pred Value : 0.6973         
##          Neg Pred Value : 0.6220         
##              Prevalence : 0.4857         
##          Detection Rate : 0.2352         
##    Detection Prevalence : 0.3373         
##       Balanced Accuracy : 0.6429         
##                                          
##        'Positive' Class : 0              
##
```

```
confusionMatrix(as.factor(dataco$nnet),as.factor(dataco$CIAF)) # Create a confusion matrix for the SVM.
```

```
## Confusion Matrix and Statistics
## 
##           Reference
## Prediction    0    1
##          0 1414  646
##          1 1421 2356
##                                           
##                Accuracy : 0.6459          
##                  95% CI : (0.6335, 0.6582)
##     No Information Rate : 0.5143          
##     P-Value [Acc > NIR] : < 2.2e-16       
##                                           
##                   Kappa : 0.2857          
##                                           
##  Mcnemar's Test P-Value : < 2.2e-16       
##                                           
##             Sensitivity : 0.4988          
##             Specificity : 0.7848          
##          Pos Pred Value : 0.6864          
##          Neg Pred Value : 0.6238          
##              Prevalence : 0.4857          
##          Detection Rate : 0.2422          
##    Detection Prevalence : 0.3529          
##       Balanced Accuracy : 0.6418          
##                                           
##        'Positive' Class : 0               
##
```

```
confusionMatrix(as.factor(dataco$RF),as.factor(dataco$CIAF)) # Create a confusion matrix for the SVM.
```

```
## Confusion Matrix and Statistics
## 
##           Reference
## Prediction    0    1
##          0 1485  544
##          1 1350 2458
##                                           
##                Accuracy : 0.6755          
##                  95% CI : (0.6633, 0.6875)
##     No Information Rate : 0.5143          
##     P-Value [Acc > NIR] : < 2.2e-16       
##                                           
##                   Kappa : 0.3453          
##                                           
##  Mcnemar's Test P-Value : < 2.2e-16       
##                                           
##             Sensitivity : 0.5238          
##             Specificity : 0.8188          
##          Pos Pred Value : 0.7319          
##          Neg Pred Value : 0.6455          
##              Prevalence : 0.4857          
##          Detection Rate : 0.2544          
##    Detection Prevalence : 0.3476          
##       Balanced Accuracy : 0.6713          
##                                           
##        'Positive' Class : 0               
##
```

##The Random forest is selected as the best model

```
setwd("D:/PhDnurtion/papers/paper 5")
library(readxl)#used to import the excel file
CIAF <- read_excel("finaldata.xls")
finaldata <- read_excel("finaldata.xls")
CIAF = as.data.frame(CIAF)
#View(CIAF)###we can see the spreadsheet of the dataset
```

```
#CIAF <- CIAF[ which(CIAF$dhs_year==4), ]
############missing value managements in the dataset
#1. identify if there is any missing values 
# 2. remove if not much
#################################### 
#is.na(CIAF$zpopulation)#toc check weather there is a missing value or not
# Recode missing values with NA.
# Replace NA by the average for the continuous variable 
CIAF$zaridity1[is.na(CIAF$zaridity1)]=mean(CIAF$zaridity1,na.rm=TRUE)
CIAF$zpopulation[is.na(CIAF$zpopulation)]=mean(CIAF$zpopulation,na.rm=TRUE)
CIAF$zpreciptitation[is.na(CIAF$zpreciptitation)]=mean(CIAF$zpreciptitation,na.rm=TRUE)
CIAF$zur[is.na(CIAF$zur)]=mean(CIAF$zur,na.rm=TRUE)
CIAF$zdrought[is.na(CIAF$zdrought)]=mean(CIAF$zdrought,na.rm=TRUE)
CIAF$zevi[is.na(CIAF$zevi)]=mean(CIAF$zevi,na.rm=TRUE)
CIAF$zirrigation[is.na(CIAF$zirrigation)]=mean(CIAF$zirrigation,na.rm=TRUE)
CIAF$zlst[is.na(CIAF$zlst)]=mean(CIAF$zlst,na.rm=TRUE)
CIAF$zmaxt[is.na(CIAF$zmaxt)]=mean(CIAF$zmaxt,na.rm=TRUE)
CIAF$zmint[is.na(CIAF$zmint)]=mean(CIAF$zmint,na.rm=TRUE)
CIAF$zpet[is.na(CIAF$zpet)]=mean(CIAF$zpet,na.rm=TRUE)
CIAF$zelevition[is.na(CIAF$zelevition)]=mean(CIAF$zelevition,na.rm=TRUE)
CIAF$zwetd[is.na(CIAF$zwetd)]=mean(CIAF$zwetd,na.rm=TRUE)
CIAF$zu5_population[is.na(CIAF$zu5_population)]=mean(CIAF$zu5_population,na.rm=TRUE)
```

```
# To improve the reproducibility of our analysis, we set the seed of the random generator 
set.seed(123456) # Set a random seed so that repeated analyses have the same outcome.
##Seeds are saved on the PC only and will not allow analyses to be repeated precisely on other machines.
index = 1:nrow(CIAF) #Create an index vector with as many sequential 
#variables as there are rows in the CIAF dataset.
testindex = sample(index, trunc(length(index)/5)) #Take a sample of 20% of the observations from 
#the index vector.
testset = CIAF[testindex, ] #Create a test (validation) dataset with 20% of the CIAF dataset.
trainset = CIAF[-testindex, ] #Create a trainig dataset with 80% of the data.
```

```
x_train = data.matrix(trainset[,c(6,7,10:73)]) # Take the features (x) from the training dataset.
y_train = as.numeric(trainset[, 74]) # Take the outcomes (y) from the training dataset.
x_test = data.matrix(testset[,c(6,7,10:73)]) # Take the features (x) from the testing/validation dataset.
y_test = as.numeric(testset[, 74]) ### based on the AUC and other statistics, the Random forest is selected 
library(ggplot2)
library(randomForest)
```

```
## randomForest 4.6-14
```

```
## Type rfNews() to see new features/changes/bug fixes.
```

```
## 
## Attaching package: 'randomForest'
```

```
## The following object is masked from 'package:ggplot2':
## 
##     margin
```

```
train_data=trainset[,c(6,7,10:73,74)] # Take the features (x) from the training dataset.
#train_data=c(train_data,stringsAsFactors = FALSE)
test_data=testset[,c(6,7,10:73,74)] # Take the outcomes (y) from the training dataset.
#test_data=c(test_data,stringsAsFactors = FALSE)
rf<- randomForest(train_data$CIAF_u5c~.,data = train_data,importance=TRUE)
```

```
## Warning in randomForest.default(m, y, ...): The response has five or fewer
## unique values. Are you sure you want to do regression?
```

```
print(rf)
```

```
## 
## Call:
##  randomForest(formula = train_data$CIAF_u5c ~ ., data = train_data,      importance = TRUE) 
##                Type of random forest: regression
##                      Number of trees: 500
## No. of variables tried at each split: 22
## 
##           Mean of squared residuals: 0.2180689
##                     % Var explained: 12.58
```

```
attributes(rf)##used to see the attributes of the model
```

```
## $names
##  [1] "call"            "type"            "predicted"       "mse"            
##  [5] "rsq"             "oob.times"       "importance"      "importanceSD"   
##  [9] "localImportance" "proximity"       "ntree"           "mtry"           
## [13] "forest"          "coefs"           "y"               "test"           
## [17] "inbag"           "terms"          
## 
## $class
## [1] "randomForest.formula" "randomForest"
```

```
rf$confusion#for example
```

```
## NULL
```
